# Detection of Predation Using qPCR: Effect of Prey Quantity, Elapsed Time, Chaser Diet, and Sample Preservation on Detectable Quantity of Prey DNA

**DOI:** 10.1673/031.009.4101

**Published:** 2009-06-18

**Authors:** Donald C. Weber, Jonathan G. Lundgren

**Affiliations:** ^1^ USDA Agricultural Research Service, Invasive Insect Biocontrol and Behavior Laboratory, BARC-West 11 1A, Beltsville, MD 20705 U.S.A; ^2^ USDA Agricultural Research Service, North Central Agricultural Research Laboratory, 2923 Medary Avenue, Brookings, SD 57006 U.S.A

**Keywords:** ladybeetle, leaf beetle, Colorado potato beetle, molecular ecology, gut analysis, *Coleomegilla*, *Leptinotarsa*, Coccinellidae, Chrysomelidae, generalist predators, biological control

## Abstract

Using quantitative PCR that amplified a prey-specific mtDNA 214 bp amplicon from the COI mitochondrial gene of the Colorado potato beetle, *Leptinotarsa decemlineata* (Say) (Coleoptera: Chrysomelidae), prey eggs of known age and number were fed to larvae of the generalist predator lady beetle *Coleomegilla maculata* (De Geer) (Coleoptera: Coccinellidae), to elucidate the effects of time and diet since consumption, number of prey eggs, and methods for sample fixation and preservation, on the quantity of target DNA detected. Signal was strongly attenuated directly after cessation of feeding, even when predators were immediately frozen at -20°C. However, the quantity of target detected was significantly related to the number of eggs consumed and the time elapsed since eating. Decrease in detected prey DNA was consistent with a negative exponential model. The target DNA sequence disappeared from starved predators (quantitative half-life estimate of 59 min) more slowly than those fed potato aphids after consuming the target prey eggs (half-life estimate 16 min), whereas those fed *C. maculata* eggs as a chaser were intermediate in the rate at which they degraded the target prey DNA sequence. Fixative protocols are of critical importance in proper use of the qPCR technique. Among seven methods tested, storing the predator immediately in 70% ethanol prechilled to -20°C yielded the highest amount of target sequence, 22.8% of that recovered directly from a single intact prey egg. Samples frozen without solvent at -80°C and -20°C yielded only 6.0% and 2.3% of the target DNA respectively, and room temperature ethanol and ethylene glycol-based antifreeze averaged below 1% recovery of target DNA. Nevertheless, target prey was detected in more than 80% of antifreeze-stored predators. Predators killed and held at room temperature for 4 h or 5 days yielded no target prey DNA in 18 of 20 cases. These results emphasize both the value and the complexities of application of the qPCR technique to field predation studies.

## Introduction

Detection and quantification of predation patterns is complex, yet essential to the development and improvement of conservation biological control (Symondson et al. 2002). Several methods have been used to determine the occurrence, frequency, and impact of predators on prey populations. These include direct observation of predation events, controlled manipulation of predator and prey numbers to determine resulting effects, and detection of prey markers in predators. Biochemical markers that are unique to the prey species, either proteins or nucleic acids, offer a versatile means for predation detection and quantification. The two leading methods are antibody-based analysis of prey proteins, and polymerase chain reaction (PCR) -based analysis of unique prey DNA sequences. Each of these techniques has advantages and disadvantages. In general, immunoassays are more expensive to develop, cheaper per sample to use once developed, and potentially able to distinguish amongst different life stages of the same prey, based on respective proteins present. PCR-based methods offer more rapid and inexpensive development (Symondson 2002) and the opportunity to simultaneously examine predation on multiple prey items, although the per-sample costs may be higher (Sheppard and Harwood 2005).

Whether or not prey DNA sequences are detectable in the predator depends on a large number of factors: choice of target sequence and particularly its length, time(s) since feeding bout(s), temperature, physiological state and mass of predator, ingestion of target or other prey or other food material before, during, and after ingestion of the prey of interest, number of DNA copies in the prey, and post-sampling preservation of the sample (e.g. freezing) to arrest target degradation. Prey DNA may be detected as a result of secondary predation (Sheppard et al. 2005 with PCR; Harwood et al. 2001 with ELISA) and/or scavenging (Juen and Traugott 2005 with PCR; Calder et al. 2005 with ELISA), which are considered false positives or erroneous detections when predation of live prey is of interest.

Conventional PCR produces a qualitative (binomial) measure of whether prey DNA is present in the gut of the predator of interest. Quantitative PCR (qPCR) reports the quantity of target DNA detected by determining how many PCR cycles are required to raise a fluorescent reporter dye above background level. This is compared with known quantities of target to obtain known threshold cycle (Ct) values that allow calculation of number of cells, organisms, or DNA copies, from tested samples with unknown content. Widely used in forensic and medical applications, qPCR has recently been adapted for predator-prey investigations. Deagle et al. (2006) examined sea lion predation on fish with qPCR detection of predator and prey DNA in fecal samples. Troedsson et al. (2007) and Nejstgaard et al.(2008) used qPCR to determine the quantities of unicellular algae in the guts of marine zooplanktonic invertebrates. Zhang et al. (2007) used qPCR to quantify the amount of *Bemisia tabaci* DNA target present in several predators in Chinese cotton fields. From these studies, it is clear that qPCR adds additional information when measuring predation compared to conventional PCR, but preliminary laboratory studies need to be performed on a study system before clear interpretations of field measures of prey consumption produced by qPCR are possible.

Controlled testing must be undertaken to define which factors have significant effects on quantified DNA target disappearance, for the specific predator-prey combinations of interest, since for both quantitative PCR and conventional PCR, differing targets (Deagle et al. 2006; Hoogendoorn and Heimpel 2001) and different predators (e.g., Greenstone et al. 2007) result in very different rates of decay in prey DNA. In the field, target quantities must be interpreted in light of feeding patterns based on predator behaviors, prey abundance, stage and availability, time of day, climate, soils, plant architecture, etc.

*Coleomegilla maculata* (De Geer) (Coleoptera: Coccinellidae) is a widespread and highly polyphagous coccinellid predator native to North and Central America. It is abundant in many agroecosystems, and is an important predator of Colorado potato beetle (*Leptinotarsa decemlineata* (Say) (Coleoptera: Chrysomelidae) in potato fields, where it may kill a large proportion of this pest's eggs as well as significant numbers of small larvae (Hazzard et al. 1991; Hilbeck et al. 1997). The dynamic interactions between *C. maculata* and its *L. decemlineata* prey necessitate the application of new tools to determine the efficacy of this predator as a biological control agent. Given that precise quantification of predation by PCR is a process fraught with complexity, a series of carefully controlled studies on this common and polyphagous predator was performed in the laboratory. Specifically, experiments were designed to determine 1) how commonly used collection/storage practices affect the detection of prey DNA within predators, 2) how prey quantity affects the outcome of qPCR analysis of predators, and 3) how time since ingestion, and the effects of subsequent feeding, affect detectability of the target prey.

## Materials and Methods

### Prey

*L. decemlineata* eggs came from a colony at the USDA-ARS Insect Biocontrol Laboratory that originated from eggs provided by the New Jersey Department of Agriculture in 1996. Field-collected insects from potato fields at the Beltsville Agricultural Research Center in Beltsville, MD, USA, were introduced yearly to maintain genetic diversity in the colony. Adults and larvae were reared using potato foliage (cv. Kennebec) as food. Eggs used for experimental feeding were fed to predators when prey was 1-, 3-, or 5-days old. One-day-old eggs were laid within 24 h of the assay, and those not fed immediately were held at 25°C for an additional 48 or 96 h respectively. Five-day-old eggs were embryonated and close to hatching. The potato aphid, *Macrosiphum euphorbiae* (Thomas) (Homoptera: Aphididae), used in the assays were collected from potatoes (cv. Kennebec) grown in greenhouses in Beltsville, Maryland, and were maintained on potato foliage until <1 h before experimental feeding.

### Predators

*C. maculata* were from a colony established in late summer 2005 collected by hand from corn and potato fields at the Beltsville Agricultural Research Center, Beltsville, MD, USA. Adults and larvae were reared under a 16: 8 (L: D) photoperiod with approximately 50% RH at approximately 25° C, and fed pollen substitute (Bee-PRO, Mann Lake Ltd., www.mannlakeltd.net), supplemented with eggs of *Helicoverpa zea* (Boddie) (Lepidoptera: Noctuidae) (Benzon Research, Inc., www.benzonresearch.com) during the early larval period, and *L. decemlineata* egg masses as 3rd and 4th instars and adults. All life stages were provided with water-saturated dental wicks.

### General feeding protocol

Fourth-instar *C. maculata* were selected for assays because they are large enough to completely consume several *L. decemlineata* eggs without becoming satiated. Before the experiment, larvae were fed on pollen substitute and *H. zea* eggs, and then were starved individually in 35 × 10 mm ventilated Petri dishes (provided with ∼8mm long upright moistened dental wicks) for 24 h prior to the start of the feeding experiment. Individual larvae were provided with *L. decemlineata* eggs of known age; times at which larvae were provisioned, began feeding, and ceased feeding, were noted to the nearest minute. A larva was judged to have ceased feeding when all eggs were consumed, and either palpal movement ceased or the larva moved from the meal site. In addition, for each experiment, a like number of replicates of unfed predator larvae, and separate groups of eggs of like age and number, were treated identically to the fed larvae when these finished feeding. Larvae not initiating feeding within 2h were discarded, and those not ceasing feeding within 4h since feeding initiation were also discarded.

### Effect of sample storage

Due to the apparent low recovery of DNA detected under the protocol of freezing at -20°C with subsequent addition of 70% ethanol at 22° C, the effect of several fixative procedures to stop DNA digestion for later analysis were compared. These treatments were selected to simulate common practices for preservation in the field or lab, to improve upon these practices, and to simulate worst-case situations with no preservation for several hours or days. *C. maculata* larvae were transferred to 0.5 ml autoclaved microcentrifuge tubes within 2 min after they had completely consumed a single 3-day-old egg. The treatments were as follows:

- egg-only positive control, using a single 3-day-old *L. decemlineata* egg, stored at -20° C in prechilled -20° C 70% ethanol (ACS/USP grade, not denatured)
- fed predator stored at -20°C in prechilled -20°C 70% ethanol
- fed predator frozen on dry ice and transferred within 1 h to -80°C freezer
- fed predator stored without solvent at -20° C for 5 days, followed by the addition of prechilled -20° C 70% ethanol
- fed predator stored at room temperature (22° C) in 70% ethanol
- fed predator stored at room temperature in full-strength antifreeze (ethylene glycol type, Prestone® Antifreeze/Coolant)
- fed predator killed with CO_2_, with addition of room temperature 70% ethanol after 4 h
- fed predator killed with CO_2_, with addition of room temperature 70% ethanol after 5 days
- unfed predator (negative control) stored at -20°C in prechilled -20°C 70% ethanol

Subsequent to fixation, samples were held at their respective temperatures: treatments 5 through 8 at room temperature; treatments 1, 2, 4, and 9 at -20°C; and treatment 3 at -80°C. All samples were processed 7 to 8 days after feeding.

### Feeding protocol: Prey egg age and quantity

Prey eggs of age 1, 3, or 5 days, were placed in groups of 1, 3, or 5 eggs in Petri dishes (n = 11–17 replicates per treatment). After feeding, *C. maculata* larvae were frozen immediately at -20° C and then transferred to 0.5 ml autoclaved microcentrifuge tubes with 70% ethanol. In this experiment, no larvae were retained after feeding, so that all samples corresponded to t = 0 after feeding. The assays on 1, 3, and 5-day-old eggs were conducted on different days, which precluded statistical comparisons of egg-age treatment levels.

### Feeding protocol: Effect of time since feeding and chaser diet

Individual *C. maculata* 4^th^-instar larvae were fed a single 3-day old *L. decemlineata* egg, and after feeding, were either fixed at t = 0 at -20° C in prechilled -20°C 70% ethanol, or held for 1, 2, 4, or 8 h and assigned to one of three treatments: starved (water wick only); allowed to feed *ad libitum* on potato aphid apterous adults, or allowed to feed *ad libitum* on eggs of their own species (eggs of unknown age from above-described *C. maculata* lab colony). Quantities of aphids or ladybeetle eggs consumed were recorded and these chaser diets were continuously replenished. At the assigned number of hours after feeding, each predator larva was killed at -20° C in prechilled -20° C 70% ethanol, and held at -20° C until processing 7 to 8 days later.

### DNA extraction

Except for designated treatments in the sample storage experiments, all samples were stored individually in 70% ethanol at 4° C until the DNA was extracted. Preliminary research (unpublished observations) showed no noticeable differences in the detectability of *L. decemlineata* DNA in whole *C. maculata* extractions versus dissected guts, and so entire larvae were extracted. Additionally, DNA was extracted from three individual *L. decemlineata* eggs aged 1, 3, and 5 days, and DNA from each group of the three *L. decemlineata* eggs were diluted independently in 1 × TE to concentrations of 0.5, 0.33, 0.1, 0.05, 0.01, 0.005, and 0.002 eggs to provide data for three sets of standard curves. Finally, the DNA of an additional 3-day old egg was extracted for use as a positive control.

Extractions were performed using DNeasy® tissue extraction kits (catalog no. 69506, Qiagen Inc., www.qiagen.com) according to product instructions. The samples were macerated in ATL buffer using autoclaved pestles, and were incubated with proteinase K for 3 h. The final dsDNA yield was quantified from each extraction using the absorbance ratio of 260/280nm (BioPhotometer, Eppendorf, www.eppendorf.com). DNA quantities from predator larvae were 30–228 µg/ml, and for individual *L. decemlineata* eggs were 6–31 µg/ml. All extractions were stored at -20° C.

### DNA amplification and quantitation

On each plate, controls consisted of three wells of unfed *C. maculata* larvae, three no-template controls, and eight *L. decemlineata* egg controls that compared the reaction efficiency among plates. In the prey age/quantity assays, a series of three standard curves for a given egg age was run along side the unknown samples; the 3-day old *L. decemlineata* egg standard curves were applied to the chaser and storage experiments. Primer sequences (fwd: 5′-CCT TTT CTC TTG GGC AGT TAT-3′; rev: 5′-TTA TCC CAA ATC CAG GTA GAA T-3′) were used to amplify a 214 bp region of the mitochondrial COI gene of *L. decemlineata* (Greenstone et al. 2007). The reaction (25 µl total volume) was composed of 12.5 µl 2X Brilliant® SYBR Green qPCR Master Mix (Stratagene, www.stratagene.com), 0.375 µl 30 nM ROX dye (as a reference dye), 300 nM of each primer, 1 µl template DNA, and 9.125 µl of molecular-grade water (Sigma-Aldrich, www.sigmaaldrich.com). Extractions were amplified using a MX3000P™ qPCR system (Stratagene) under the following conditions: 95° C for 10 min, followed by 50 cycles of 95 C for 30 s, 54° C for 1 min, and 72° C for 1 min. Fluorescence was recorded at 492 nm during the annealing step of each cycle. To ensure that only the desired product was amplified, a dissociation curve was produced for each reaction by heating the samples to 95° C for 1 min, then dropping the temperature to 55° C and ramping up at 0.2° C/s to 95 °C, and monitoring fluorescence continuously. The PCR product of this reaction dissociates unimodally at 74.15° C.

### Data analysis

The fluorescence threshold was adjusted manually to bring the dRn (baseline-corrected normalized fluorescence) just above background fluorescence that ranged from 0.01–0.05 units. For each sample, the resulting cycle at which the fluorescence could be observed above background (Ct) was exported to a spreadsheet. Reactions that did not amplify within 50 cycles were considered non-detected. The response variability among the plates was controlled for by taking an overall mean value from the 24 positive controls; Ct values for the 1, 3, and 5-day old egg treatments were standardized by -0.94, 0.16, and 0.79 units, respectively. A standard curve of Ct values for the three sets of known *L. decemlineata* egg dilutions was generated for each egg age class and its significance was determined with ANOVA (SAS Institute 1998). Higher-order polynomials were tested for significance; the resulting linear regressions were used to determine unknown egg equivalent detections corresponding to the reported Ct values.

The effect of fixative treatments was compared using three criteria: how many samples had detectable target DNA of any quantity (within 50 cycles); how many prey egg equivalents were detected; and how much total DNA (predator plus prey) was present in the sample (µg/ml). Overall tests for treatment effect amongst the seven fixative protocols were performed using a *χ*^2^ test for percent detected and Kruskal-Wallis H statistic for egg equivalents and total DNA (SAS Institute 1998). Subsequently, planned single degree-of-freedom nonparametric orthogonal contrasts were used with Fisher's exact test for percent detects, and with Mann-Whitney U test for egg equivalents and total DNA (SAS Institute 1998) to detect differences between individual treatments, and logically-grouped sets of treatments, and positive and negative controls. For samples where *L. decemlineata* was not detected, the values were randomized between zero and a conservative estimate of the detection threshold for the purposes of statistical comparisons. This detection threshold corresponded to Ct = 42, the minimum whole number of cycles above which no target was detected for the fixative experiment dataset.

The effect of elapsed time with three chaser diet treatments was tested using non-linear covariance modeling (SAS PROC NLMIXED, SAS-STAT v9.1, SAS Institute 2003; Milliken and Johnson 2002). A three-parameter negative exponential model was fitted to the curves using the common (pre-chaser) data at t = 0 and treatment-specific data for post-feeding time elapsed. Initially, the full model with all three parameters in the equation y = α + βe^-γt^ was applied independently to the three chaser treatments. In this three-parameter model, α is the asymptotic minimum as t → ∞; β determines the starting value when t = O, and γ is the exponent which models the rapidity of decay. Using SAS PROC NLMIXED (SAS Institute 2003), the model was subsequently simplified when parameters were not significantly different among treatments. The effect of observed consumption of chaser diet (aphids or *C. maculata* eggs) on egg equivalents detected was tested using ANOVA (SAS Institute 1998) within chaser treatment and time elapsed.

## Results

### Effect of egg number on target sequence quantity

**Figure 1.**
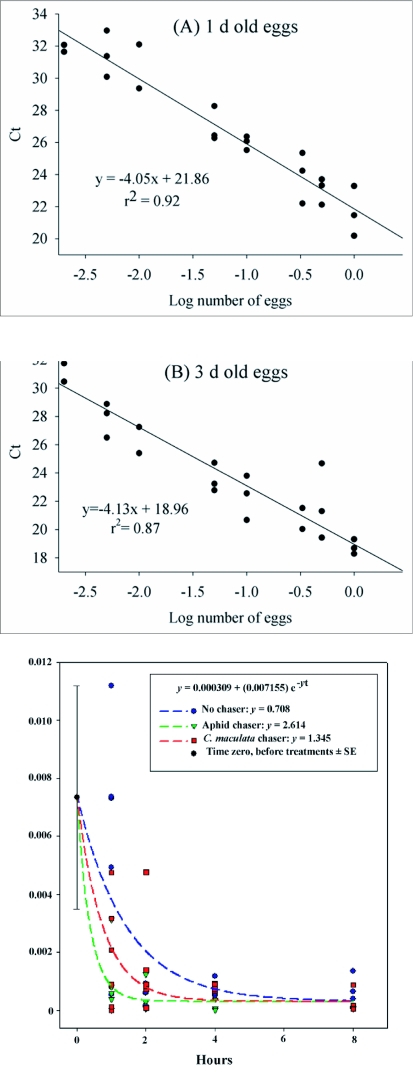
Standard curves relating the number of *Leptinotarsa decemlineata* eggs versus Ct, or threshold cycle, for eggs aged 1 (A), 3 (B), and 5 (C) days.

**Figure 2.**
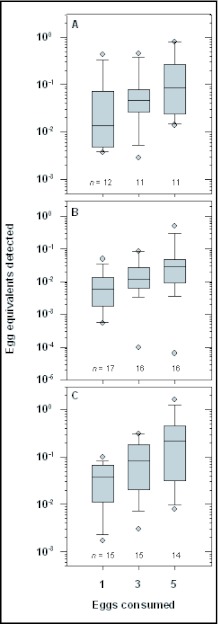
*Leptinotarsa decemlineata* prey eggs detected by qPCR, versus eggs consumed by *C. maculata* predator, immediately at cessation of feeding (t = 0), for eggs aged 1 (A), 3 (B), and 5 (C) days. The box plots show median, 25^th^ and 75^th^ percentiles, with whiskers at 10^th^ and 90^th^ percentiles and individual data points shown outside this range.

For all three egg ages, the log number of eggs consumed had highly significant effects on Ct values. The separate within-age-class standard curves are presented in Figure 1; all three produced significant linear regressions (p<0.0001), with no significant higher-order terms. These curves were used to determine egg number detected in experimental samples. All 127 assays at t = 0 were successful; however, the quantities detected were very much less than that recovered from one intact prey egg, and also showed variability in quantity (egg-equivalents) detected at t = 0. Egg number consumed significantly affected the amount of target DNA sequence detected (p <0.0001; F_(1,125)_= 18.74 for log(x+1) transformed egg equivalents) (Figure 2). The proportion of egg-equivalents detected (egg-equivalents detected divided by eggs fed) did not differ by number of eggs fed for any egg age class (p = 0.59, F_(1,32)_ = 0.29; p = 0.56, F_(1,47)_ = 0.35; p = 0.21, F_(1,42)_ = 1.65; respectively for eggs of age 1, 3, and 5 days). This indicates that the quantification is proportional to eggs consumed, for equal egg age. Proportion of egg-equivalents detected also did not differ by duration of predator feeding (p = 0.56, F_(1,32)_ = 0.36; p = 0.45, F_(1,47)_ = 0.59; p = 0.30, F_(1,42)_ = 1.08; respectively for eggs of age 1, 3, and 5 days).

### Effect of fixative on target sequence detectability, quantity, and total DNA

Fixative protocol, how a sample was treated at t = 0 after the predator had just finished its meal, had a highly significant effect on percent detection, egg equivalent detected, and total DNA (predator and prey) present in the sample (Table 1). Detection ranged from 100% to 0%, based on an average n = 10 ± 1 replications. Of the target DNA present in the intact egg (positive control, treatment 1), the maximum detected in predators was significantly less, 22.8%, for the highest recovery, the -20°C ethanol treatment (treatment 2). Total DNA in fed predators (treatment 2) significantly exceeded that found in unfed controls (treatment 9), even though the total DNA in the intact egg (as measured for treatment 1) was less than 10% of the total DNA detected in the fed predators (treatment 2). Warm, dry predators killed by CO2 and held for 4 h or 5 days at room temperature (treatments 7 and 8 respectively) had similar detection frequencies to the unfed controls (treatment 9).

Single-degree-of-freedom nonparametric orthogonal contrasts were used to test the differences among logical groups and individual treatments. As a group, frozen samples were significantly better preserved than were those held at room temperature (22°C), as measured by all three criteria. Within the three frozen treatments, freezing the sample with ethanol (-20°C), resulted in significantly higher egg-equivalents detected (mean >5-fold higher than dry frozen treatments). Freezing with ethanol, however, did not result in higher detection frequency or total DNA which were high for the whole group of frozen treatments. Comparing dry freezing at -20°C and at -80°C, there were no significant differences in any measure. Within room temperature treatments, solvents (ethanol and antifreeze) significantly improved percent detection and egg-equivalents detected, but did not show greater total DNA. Examination of room temperature solvent treatments in more detail showed that while ethanol and antifreeze did not differ in detection frequency or egg equivalents detected, the antifreeze treatment significantly reduced overall DNA. Within the warm dry killed predators for 4 h or 5 days, both detection frequency and quantity did not differ significantly as both were low to nil, and there was also a significant reduction in total DNA in the samples held 5 days compared to 4 h.

Combining cold temperature with solvent, the fixative protocol that preserved target DNA best was ethanol at-20°C. Predators placed in -20°C ethanol were rapidly killed, becoming rigid without movement within < 1 s. Although an average of somewhat less than one-quarter of ingested target DNA was recovered in these predators, this exceeded the next-best treatment, dry freezing at -80°C, by over three-fold.

### Effect of chaser diet and time since feeding on quantity of target DNA sequence

**Figure 3.**
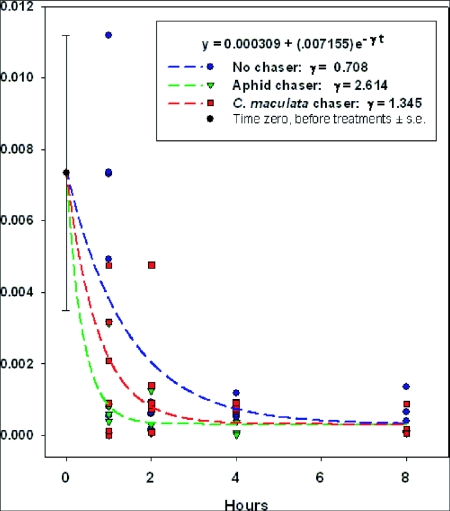
Chaser effect on quantity of detected *Leptinotarsa decemlineata* eggs detected by qPCR in *C. maculata* 4th instar larvae at 1, 2, 4, and 8h after consumption of a single *L. decemlineata* egg at time zero.

The three-parameter negative exponential equation significantly accounted for time effect on quantity of target DNA remaining, and there was a significant effect of chaser diet on the quantity of target DNA over time (Figure 3). Not unexpectedly because of the low to zero values at t = 8 h and with common t = 0 pre-chaser controls, parameters α = 0.000309 and β = 0.007155 respectively, did not differ by chaser treatment (Figure 3). The exponential parameter γ, which determines the rapidity of decline over time, significantly differed over chaser treatment (overall F_2,66_ = 19.04, p <0.0001) such that starved predators had higher levels of target DNA sequence over time than aphid-fed predators (comparison F_1,66_ = 37.07, p <0.0001). Predators provided with *C. maculata* eggs as chaser diet did not differ significantly from either of the other treatments (p >0.17). The half-life, after which half of the quantity present at t = 0 is expected to disappear, was calculated as t_1/2_, = -ln(0.5)/γ. Thus using the γ values (Figure 3), the estimated half-lives for quantity of *L. decemlineata* target sequence were: 59 min for starved, 16 min for aphid-fed, and 31 min for *C. maculata*-fed predator larvae. All predators provided with chaser diet ate at least some aphids or *C. maculata* eggs (for 8 h, 7.30 ± 1.92 aphids, or 25.1 ± 16.5 eggs), but there was no significant effect of chaser consumption within treatment and elapsed time class (all eight cases, p >0.11).

## Discussion

Attenuation of DNA signal as detected by qPCR upon ingestion was rapid, substantial, and variable, but the quantity ultimately detected was related to the amount of prey consumed. This reduction in detectable DNA occurred within minutes of the predator larvae ingesting 1, 3, or 5 *L. decemlineata* eggs. Using the initial preservation technique of dry freezing at -20°C a range of 0.00128–43.60% of DNA ingested was detectable, representing a 2.29 to 78,125-fold attenuation of signal. The rapid decay in target DNA even before t = 0 post ingestion was also seen by Nejstgaard et al. (2008) in copepods, in which qPCR recovery at t = 0 for ingested unicellular algae were compared with positive qPCR controls (intact prey organisms, as in our study), and also using microscopy and chlorophyll fluorescence. Recoveries at t = 0 “were consistently lower than expected”, from 2 to 32% of the estimates provided by fluorescence readings, and from 11–20% of those provided by classical microscopy. Nejstgaard et al. (2008) examined three possible sources of this large reduction in signal: inhibition by predator DNA, post-treatment freezing, thawing and refreezing (part of their post-feeding protocol), and rapid digestion by the copepods, which available evidence suggests have digestion rates comparable to those of insects. Their conclusion was that interference by predator DNA was unimportant, at least at ratios of less than 10,000:1 predator:prey DNA. In our system the predator: prey ratio of DNA was estimated at less than 30:1, and concerns that inhibition may occur (Juen and Traugott 2006) are negligible in our case.

**Table 1.**
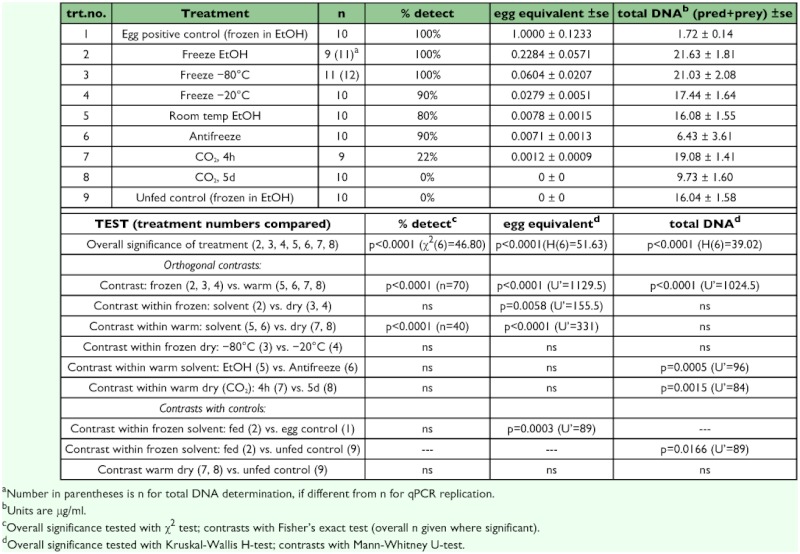
Effect of fixative protocols on detection of prey DNA, quantity of prey DNA detected (in egg equivalent) and total DNA in sample (including predator and prey). Prey is a single CPB egg, fed to *C. maculata* larva, fixed immediately after cessation of feeding.

Digestion in the coccinellid tested, apparently even during a meal, is very rapid. The predator's digestive system aggressively degrades the prey, but in common with other studies (Zhang et al. 2007; Nejstgaard et al. 2008), the amount of prey that was detected at t = 0 did reflect how much prey was consumed. The rapidity of target degradation in coccinellids and several other coleopteran predators tested implies that positive detection using conventional PCR, and qPCR-detected prey quantities, could easily vary according to the predator population's diel pattern of feeding such that sampling at different times of day would yield contrasting assessments of predation. The exponential decay pattern in quantity of target DNA detected by qPCR in predator guts following ingestion is consistent with the expectation that a constant proportion of target disappears per unit time. Exponential decay has also been found in many instances with quantitative antigen detection using ELISA (Lövei et al. 1985; Sopp and Sunderland 1989). In the case of conventional PCR, target DNA decay rates are not available since the data are presence/absence. Logistic models can be used to characterize the decline in detectability, including the so-called detectability half-life (Greenstone and Hunt 1993 and Sunderland 1996 for antibodies; Chen et al. 2000 and Greenstone et al. 2007 for PCR).

The predator's diet subsequent to ingestion of target prey has been shown to affect detection of target prey marker by immunoassay (Symondson and Liddell 1995, Fournier et al 2006) but not by published studies using conventional PCR, so that our result with qPCR, though novel, is not unexpected. Both chaser prey (e.g., Hagler and Naranjo 1997; Symondson et al. 1999; Greenstone et al. 2007; Harwood et al. 2007) and starvation (e.g., Hagler 1998; Chen et al. 2000; Harper et al. 2006) are routinely used in studies to determine rate of target prey marker disappearance. Hed et al. (1999) found that a chaser diet of aphids versus apple for the coccinellid *Hippodamia convergens* influenced the proportion carrying the fungal pathogen *Discula destructiva* (dogwood anthracnose) and excreting viable spores in their frass.

Our tests of several widely used fixation protocols also show that qPCR results are extremely sensitive to the method of fixation and preservation of samples. Over 99% of DNA is easily lost through techniques in widespread use, such as placing samples in warm ethanol, dry cooling or freezing at unspecified temperatures. All of these practices appear to allow the aggressively destructive process of prey digestion to continue with surprising rapidity. We have also observed that both dry freezing and room-temperature ethanol may result in regurgitation; this occurred, for instance, in 31 of 120 instances for our predators frozen dry at -20°C. These results suggest using -20°C pre-chilled 70% ethanol as the fixative/preservative of choice. However, even colder temperatures and possibly different solvents may further improve the detection level in genetic gut content analysis. For instance, preservation of tissues for medical and physiological research typically specifies so-called snap or shock freezing in isopentane that is either cooled by suspension in liquid nitrogen or by addition of dry ice (Mager et al. 2007).

To be of use in studying predation dynamics, quantitative PCR requires calibration including estimation of many interacting factors. The first challenge is to minimize unnecessary variation and biases in data collection, such as those caused by ill-advised bulk sampling (see Harwood and Obrycki 2005) or less-than-optimal fixation of samples. The more complex challenge is to use ecological experimentation and observation to provide useful estimates of important factors such as pre- and post-ingestion diet, temperature, etc., and to devise the appropriate models to integrate these factors, so that the results provide answers regarding predation processes occurring in the field.

## **Abbreviations**

COI: cytochrome c oxidase subunit I,
qPCR: quantitative PCR
